# Von den Krankheiten des Menschen. Allgemeine Pathologie

**Published:** 1843-04-01

**Authors:** 


I. VON DEN KRANKHEITEN DES MENSCHEN. ALLGEMEINE PATHOLOGIE. Von Dr. *K. G. Neumann*. Berlin, 1842.

On the Diseases of Man. General Pathology.

II. ELEMENS DE PATHOLOGIE GENERALE. Par *A. F. Chomel*. Paris, 1841.

Elements of General Pathology.

WE have now lying before us some half-dozen works, published within the last four or five years in France and Germany, on the important subject of general pathology, scarcely any two of which agree with respect to that which should constitute this science. Some have treated it as if they considered it identical with Semeiology; some have made it to consist almost exclusively of scraps of pathological anatomy. Whilst others, and these by far the most numerous, have introduced into their treatises abstruse and metaphysical discussions regarding the various occult causes and nature of disease, discussions which should be altogether excluded from works intended for the perusal of students. The legitimate object of a work on general pathology is the study of diseases in the abstract, and in that which they have in common. It should serve as a complement to special or descriptive pathology, just as general anatomy does to descriptive anatomy. It should comprise all that is most simple and most elevated in science, on the one hand, the definition of the terms, and the description of the phenomena of diseases; on the other hand, the discussion of all the fundamental questions, and the exposition of the general principles which should serve to guide the physician in the arduous exercise of a profession so intimately connected with the dearest interests of humanity. General pathology, in fact, includes within itself the humblest elements and the most sublime philosophy of medicine.

Dr. Neumann, the author of the work mentioned at the head of this article, is well known all over the Continent by his medical writings. The work of his now before us he divides into two sections; the first treats of the Generalities of Disease, viz. the course, causes, symptoms, duration, &c. of disease—the second, of Diseases of particular Systems, as of the vascular, muscular, lymphatic, &c. systems.

We shall here pass over a great deal of what our author says on the causes and course of diseases, as being quite uninteresting to our readers, and proceed to the consideration of diseases of the arterial system. These he divides into those affecting the structure, and those affecting the motion of arteries: he first commences with *ossification*: this change of structure he observes increases with age; in persons under thirty years it is seldom observed; whilst in men beyond seventy it is seldom absent. Wherever such changes occur, they diminish the nutrition of the parts to which the arteries lead; nay, they ultimately suspend it altogether, and occasion a peculiar species of gangrene, not preceded by any inflammation whatever, but merely by a sense of weight in the gangrenous part, and impeded motion; this occurs in the aorta and in the arteries of the lower extremities. The coronary arteries of the heart are most prone to ossification.

Such is the account, and the entire account, given by our author of so important a subject as ossification of arteries is admitted to be. Not a syllable does he tell us with respect to the causes of ossification. Perhaps, however, he was prudent in observing silence on a matter of which so little is known. The partisans of the general theory of irritation will have it, that ossification is the result of inflammation of the arteries. We know that obliteration of these vessels is oftentimes produced by inflammation; and this is so evident, that we may artificially produce this obliteration by exciting inflammation. But the case is entirely different with respect to ossification of the arteries. It has been urged, but without sufficient grounds, that the arteries most frequently ossified are precisely those most frequently irritated; such is not the case; it is not true that the arteries of the lower extremities are more frequently irritated than those of the upper. All the suppositions advanced on this subject are entirely erroneous. The fact is, we know nothing whatever of the causes of arterial ossification: all we know is, that aged persons are more subject to this morbid change than young persons, and males more than females; but beyond this our knowledge does not reach.

The next question which presents itself to the consideration of the pathologist, and which our author does not even salute, is, how this ossification is established? what are the parts primarily affected? According to Bichat, it is the internal coat which presents the first traces of ossification; this is also Mr. Guthrie's opinion, an opinion, moreover, which he supports by several specimens of morbid anatomy. It has been stated, however, of late years, that the ossific deposits or plates are first found between the internal and middle tunics; others, in fine, think with Morgagni, that the disease commences by the middle tunic; be this as it may, after a certain time, the middle tunic is always affected, sometimes by the formation of small osseous scales, and sometimes by prolongations of a fibrous appearance.

If the manner in which the osseous laminæ of the arteries have been formed has been thus controverted by authors, there has been no less difference of opinion with respect to the nature of these osseous products; it has been asked, in fact, whether they are to be attributed to a simple deposition of calcareous matter, to a mere inorganic superposition between the arterial tunics, or whether they are organized like bones. The first question has been answered in the affirmative by Hodgson and Beclard, who however made some exceptions; Morgagni, on the contrary, admitted that it was the same with the ossification of the arteries as with that of the falx cerebri; that, in the two cases, there is a real organization, which appears probable enough; since the two products, when treated by chemical processes, yielded about a third of animal matter, with two-thirds of phosphate of lime.

Though we can hazard nothing but mere conjecture regarding the causes of arterial ossification, and with respect to its mode of production, one part of the subject, at least, we know full well, namely, the fatal effects produced in the system by this species of degenerescence. These effects are local and general. The principal local effects are, inflammation, rupture, contraction, obliteration, ulceration and erosions of these vessels; aneurysms, in some cases, have been referrible to this cause. The general effects produced by ossification of the arteries are very numerous. They may result from ossification of the aorta; now, it is evident that ossification of this vessel must introduce serious disturbance into the circulation, and thereby into all the functions of the œconomy. Some go so far even as to say that to this morbid change must be attributed the state of *decrepitude* observable in aged persons, and even to consider all cases of premature old age as the result of calcareous incrustation of the arteries.

There are two other states attributed, and with good reason, to ossification of the arteries, viz. atrophy and gangrene. The heart is found atrophied in cases of the coronary arteries being atrophied. Some have supposed that the diminished size of the brain in some individuals might depend on the basilar and internal carotid arteries being ossified. Gangræna senilis has been attributed to arterial ossification.

Our author next notices dilatations of the arteries and aneurysms; on this subject he says, that "either the artery, as the aorta for instance, becomes extended, or the external membrane of the artery gives way, and the inner tunic escapes from the fissure in the form of a sac: this constitutes *aneurysm*. The places where these dilatations occur most frequently, are the arch and origin of the aorta, more rarely the descending aorta; the iliac, femoral, popliteal and brachial arteries. They may occur also in other places, and occasionally entire arteries degenerate in such a manner, as to make their appearance at the same time in several places."

This, we must confess, is a very meagre account of so important a morbid change as aneurysm, in a book especially devoted to general pathology. Aneurysms have been conveniently divided into *spontaneous* and *traumatic*, the former arising from internal causes and the latter from external injury. Spontaneous aneurysms have been again subdivided into *true* and *mixed;* in the former case there is more or less dilatation of the three tunics at once, without rupture or laceration of any. Sometimes there is only dilatation of the external or cellular tunic, with rupture or destruction of the internal or middle tunics; this has been styled *external mixed aneurysm;* whilst sometimes it is only the internal coat which is dilated, and escapes through a rupture in the middle coat.

Our author entirely overlooks the very important subject of inflammation of arteries, contraction or obliteration of these vessels, and next proceeds to consider the pathology of the venous system. The veins, he says, are also subject to peculiar diseases. Their vitality is, no doubt, rather low, for they are devoid of sensation, and one would be less in error in saying that they were passive than in predicating the same thing of arteries. In the next place the centre of the vascular system has much less influence on them than on the arteries. Still we not infrequently hear mention made of the venous temperament, of the venous constitution, of venous diseases of all kinds. They are capable of becoming inflamed, of passing into suppuration, and of thereby occasioning death; foreign substances also, when introduced into them and carried to the heart, soon kill, even things apparently of the greatest indifference, viz. a little atmospheric air, a small portion of cruor taken from any animal. In cases of venous inflammation, if the pus reaches the heart, it also proves fatal. They are capable of dilating and contracting to a high degree, so that their capacity for the mass of blood becomes very rapidly changed. Mere external warmth, muscular motion, the dependent position of a limb extends them, the opposite conditions produce great and rapid change in their calibre. But we cannot with perfect certainty estimate the consequences of all this with respect to the production of disease; we can only suspect them. They are sometimes locally distended, become swollen and form knots, small vessels on these knots often degenerate and assume the appearance of veins. Varix is accordingly more a disease of these than of the veins, which swell still more rarely than the arteries. So that there are spurious varices as well as spurious aneurysms, that is, if we give the name of true varices to those, where the venous trunks become swollen; these are almost as rare as true aneurysms, and almost all varices are spurious, viz. swelling of the small vessels with a conversion of the same into venous structure.

What share the veins take in the motion of the blood contained in them, is not easily determined. The suction-power of the right side of the heart acts immediately on the blood of the cavæ; but does this suction-power also act on the small vessels? To this may be added the great irregularity of the motion in the veins, which is sometimes rapid, sometimes slow; namely, according to the variety of the muscular motion or rest of the body. The most difficult point of all to explain is the acceleration or retardation of the motion of the blood in the veins through mental emotions, which yet is so very striking, that fear or terror, for instance, as it were freezes the blood in the veins, and the open venous trunks, after such affections of the mind, do not pour out a single drop of blood, whilst joy or anger is capable of accelerating the stream of blood into the veins in a very high degree. It is certain that this effect is produced through the ganglionic nerves, but it is not probable that these nerves have no influence on the venous trunks, the veins being destitute of nerves. These nervous impressions may act immediately on the heart and arteries, and mediately on the small vessels; but this affords no satisfactory explanation of this phenomenon, so common and so well known, though still so concealed as to its cause.

The great variety of structure of the veins in several organs leads one to suppose that they will act very differently, and comport themselves very differently in disease, and experience fully bears us out in this supposition. The cranial cavity contains a venous system, to which none other is similar; contrary to the nature of all other veins, a provision has been made to render it entirely incapable of dilatation or contraction. It is therefore lodged in the tense dura mata, where this membrane is most immoveable. For this purpose frequent communications open from it externally, so that the current and passage of the blood goes on without any impediment, and pressure by extension on the brain is impossible. Thus has Nature in her wisdom protected the noblest and most important of her structures by surrounding it with a net-work of soft veins, which are capable of drawing off the blood from the cerebral substance, a thing which hard veins could not effect. However, if the return of the blood through the jugulars be impeded, the consequences are distress, stupor, redness or lividity of the face, and watery swelling of the eyelids. If, by repeated pressure of the blood on the brain through the abuse of narcotics, the diameter of the soft veins of the inner membrane becomes gradually dilated, this disposes to various diseases of the brain. With respect to the veins of the abdominal cavity, a different order of things presents itself from that of the other veins: they unite into one trunk, which, distributing itself to the spleen and liver, continues soft in the former, whilst in the latter it divides itself into branches, the membranes of which are almost as solid as those of the membranes of the sinuses, and resist all extension. These branches again collect themselves into one trunk, which opens into the inferior cava, from which the blood can reach the vena portæ just as easily as it runs from the latter into the cava. And this is the arrangement of this vessel, whence the circulation in the abdomen is much slower and more uncertain, than in any other part. Hence also the disposition to congestions of blood in the abdominal veins and in the spleen, the organ which admits the blood of the abdomen, when the liver is filled: hence the full state of these veins, in cases of threatening of suffocation, or after death by suffocation, wherein the intestinal membranes are observed to be covered over with red spots; hence the proneness to hæmorrhages from the spleen and stomach. A number of phenomena, which persons are wont to refer to sanguineous repletion of the abdominal veins, have entirely different causes, viz. hæmorrhoids, gout, hypochondriasis, melancholy, &c.

The minute vessels constitute the most important part of the entire vascular system, in as far as the end of the whole system is the support of the vegetative process, and as this is peculiarly the business of these small vessels. It may be that the anatomist sees in them mere continued arteries, or mere beginnings of veins, and is merely perplexed respecting the limits of both systems because he sees none; for the physiologist and pathologist there is a decided difference between these small vessels and arteries and veins. For to him the blood does not circulate for the sake of circulating, but in order that from it all the parts may be nourished, all the fluids may be secreted, which takes place neither in the afferent nor efferent vessels, but in the minute vessels. The physiologist and pathologist know that the small vessels have an influential atmosphere, and that they extend their power of sustaining vegetation beyond their physical limits, as, to use an obvious example, is clearly proved by the connexion between the maternal and fœtal placenta. These find themselves at no loss concerning the limits of the arterial and venous systems; for they consider all the arteries to terminate gradually in small vessels, from which all the veins commence just as gradually. These minute vessels constitute with them a system entirely distinct from the afferent and efferent vessels, which has its own peculiar life, and also its own peculiar diseases. Their investigation is no doubt beset with considerable difficulties; for, as almost all the organs are composed of minute vessels, the diseases of these appear in fact as special forms of diseases of compound parts, and the problem of the pathologist is, from the special phenomena to select those which appertain to the minute vascular system. The problem which we have now before us is, accordingly, to investigate to what forms of disease the individual organs are obnoxious, especially with respect to their vascular structure. Four points come here under consideration, *congestion, inflammation, hæmorrhages, spasm.* (Krampf.)

Once we have succeeded in establishing what inflammation is, it will appear how possible it is, that organs whose vessels are not demonstrable should however become inflamed; the crystalline lens is an instance of this. The serous membranes have vessels and become inflamed, oftentimes with increased effusion: vascular structure was for a long time denied to them, but their inflammation could not be doubted, and this must be always considered as effected by the minute vessels. The theory of congestion is difficult to be separated from that of inflammation, as both notions become clear only by comparison. The idea of congestion is in fact simple; if the blood accumulates in the minute vessels of any organ whatever, so that it dilates its vessels in diameter, and thereby occasions a change of colour and circumference of the same, without however producing any change in its normal state of vegetation, this state of things we call congestion. The words "without however, &c." must not be taken in too strict a sense: for certainly the secretion in such an organ is greater, and consequently the process of vegetation in it goes on differently from what it did in the state of health. But it maintains its original form, and the interchange of substances continues in it.

" Congestion is divided into arterial and venous, according as the accumulation of blood is occasioned by greater influx from the afferent vessels, or through impeded return through the efferent vessels. This division is important but not adequate; for there is a third species of congestion which depends on immediate irritation of the place in which the blood is accumulated. This irritation may be of a very various kind; *e. g.* mere nervous irritation, an idea which is reflected on a ganglion occasions it, just as a blush on the cheek of a person taken by surprize, or it is the consequence of a mechanical or chemical external action, as friction of the skin, the application of mustard," &c.

Here we have neither changed arterial nor changed venous action, and yet there is congestion. It is also justly divided into active and passive. In every congestion the extension of the vessels of the part where it is, is increased, but this increase may be a consequence of exalted action of the extending power, or else the effect of the diminished contracting power; the former is active, the latter passive. If, *e. g.*, parts swell from external cold, or if this occur after concussion, a fall, &c., it is passive congestions have occasioned this, with diminished contractility, and accordingly apparently exalted expansion without exaltation of the expansive power. All local irritations afford examples of active congestions.

But when the predominance of expansion over contraction so changes the vegetative process in an organ, that it swerves from its normal state not only in degree, but also in kind, and consequently the nutrition of the swollen structure continues no doubt, but with an essential change in form, we then pronounce it to be *inflamed*. Increased expansion is not merely confined to inflammation, as it may be even greater in mere congestion than in inflammation. Nor is a change in the vegetative process confined to it, as that may occur without any inflammation, as in the case of scirrhus, tumors, &c.; but it is essential to it, that the process of vegetation should be changed by predominance of the expanding power. Hence it at once appears, how the organs of vegetative life, which have neither nerves nor vessels, are yet capable of coming into a state analogous to inflammation, namely, by expanding and at the same time changing their vegetative process. It is possible, nay, it is even probable, that anatomy will at length succeed in demonstrating vessels in the crystalline lens, the only organ, which is inflamed, without having blood-vessels, just as it has succeeded in the case of serous membranes. The organs capable of the greatest expansion are the minute blood-vessels; consequently it is through them that inflammation is essentially effected. But as they every where stand in the closest connexion and in a sort of antagonism with the vessels and nerves, the latter take a share in the inflammatory process, so that during it they are pressed and rendered tense by the organic substance in the part affected, and consequently must suffer in proportion as the inflammation increases. It is true that inflammation properly so-called is only possible where vessels and nerves unite into one structure; where this is not the case, we can only speak of a state somewhat analogous to inflammation.

Our author next proceeds to draw a line of demarcation between inflammation and congestion: congestion, according to him, is to be distinguished from inflammation, not by the degree of extension, for it may be very great in the former and very small in the latter, but by the change occurring in the plastic process. By this it is not asserted, that this process goes on perfectly normally in organs which are the seat of congestion; we must rather acknowledge that every change of action in the productive system is necessarily followed by change in the product, and accordingly that during the congestive state the organ must secrete otherwise than in the healthy state. But the product is only increased in degree, only inconsiderably modified; it still follows the same type, the same standard, which it followed in the state of health. But not so in inflammation; this changes the crasis, form, and action of the organ entirely. An example will explain the matter: we shall take it from the skin, the organ whose changes fall most under the cognizance of our senses. If the peripheric action of the vascular system be great, or if from violent muscular exertion the skin perspires, it becomes redder and warmer than at other times, it secretes much more copiously, nay more, the matter secreted is different in quality from that ordinarily given off. No doubt congestion of blood to the skin takes place, but the vegetative process of the same organ is not in the slightest degree affected; the skin remains skin; it continues to receive nutrition according to its usual type. But suppose, the skin is covered in any part with a blistering plaister, inflammation takes place on its surface, that is, in case of much less congestion, the relation of the plastic process between the skin and epidermis is changed; the latter disengages itself from the skin, and the interval is filled by a serous secretion. Or suppose, a phlegmonous inflammation of the skin itself takes place, the ordinary secretion of this organ ceases entirely, its substance swells, becomes loose, thick, deep-coloured, and in a few days the vegetative process is so much altered in it, that it is changed into the fluid form, and separates from the living part. * * * * * *

On the Degeneration of Muscular Substance our author says:—Degeneration of muscular substance takes place in the course of life in many ways. It is commonly said that through age alone it becomes hard and dry, but that is true only to very limited extent; the cellular tissue, the membranes shrink much more in the course of years than muscles. But inactivity or disuse changes the muscular substance either into fat, whereupon motion then becomes entirely impossible, or it forms from the broad muscles mere membranes, and, from the long muscles, thin, shrivelled fleshy bundles. In a similar manner too violent exertion changes the muscle; the tendon increases gradually at the expense of the flesh, and the latter becomes harder, more torpid, and less fitted for motion. The very reverse of this torpidity we observe to occur many times, especially after diseases; the fibres of the muscle appear to go from one another, the cellular tissue connecting them seems to become more flabby and to contain a considerable quantity of serous fluid. If a muscle be divided in this state, serum exudes after the bleeding is over. In the case of animals fed under the care of man, this state of disease sometimes occurs unexpectedly; they then approach the dropsical state. Should this watery swelling be considered as exudation, the mistake will be obviated by the fact that no inflammation preceded. This state of the cavities or of the cellular tissue is not always preceded by dropsy, but yet dropsy seldom comes on without the muscles being then affected. Individual muscular fibres often separate, become torn, connect themselves with others, and so give rise to pains, the causes of which are difficult to be traced. Steatomatose formations sometimes occur in it, as also hydatids, always however only singly, and oftener in animals than in man.—What has been said here holds good only of the voluntary muscles.

The tendons hold a very low place in the scale of vitality: they never inflame, do not swell, do not become dropsical, take no share in the diseases of the limbs to which they belong till a late period, they withstand mechanical force in a high degree, and are scarcely capable of any other form of disease, than what they suffer through mechanical injuries. In such cases they do not swell, and they unite but imperfectly by cellular substance, which assumes considerable strength. Tendons may become ossified in old age.

We next come to the *Lymphatic System*. After considering this system anatomically and physiologically, he next views it in a pathological point of view. The lymphatic system is proved to stand at a very low ebb of vitality, a circumstance which no doubt must influence it more or less when attacked by disease.

Still the lymphatic vessels are susceptible of disease; they are liable to inflammation, and in that state are capable of exciting considerable pain. Poisoned wounds cause pain in the lymphatic vessels which have taken up the poison, and in the glands to which they lead. How acutely do not buboes pain, and the axillary glands in cancerous breast? The inflamed lymphatic vessels are sometimes seen to run along beneath the skin. Inflammation runs along them, as in all organs of low vitality, in a very tedious course, until it is dispersed or passes into suppuration, which occurs but seldom. The lymphatic glands, without any very remarkable degree of inflammation being perceptible, are capable of a great increase of size, the interior glands of the mesentery less than the exterior, which sometimes attain a really monstrous size, and retain it for a long time without destroying the individual. Still the mesenteric glands do reach sometimes a considerable size; in this case the diameter of the vessels conglomerated within them becomes dilated, and these glands are incorrectly said to be obstructed, as they are much more pervious than in the healthy state, as quicksilver injections reach such glands most readily. This swelling of the glands very frequently occurs in children, and then continues very often for the entire course of childhood, until the period of adolescence, when it disappears.

According to the writings of several pathologists, the lymphatic system is the seat of almost all those diseases usually referred to that state of the system connected with dyscrasia, and performs a very important part in the production of disease. But if we compare these assertions with the general properties of this system, according to which the lymphatic vessels take up everything presented to them almost mechanically, and want a central point to determine their action; as also with the phenomena themselves, from which it appears that their glands may be for many years in a state of disease, without the individual dying, though he may suffer more or less, especially in his nutrition; if we compare with this the undeniable facts that the most formidable poisons may remain for a long time in the lymphatic system, without occasioning any mischief whatever, but which will at once appear, if they reach the sanguineous system, considerable doubts must arise regarding the correctness of the assertions made by pathologists. The following facts are established:

*a.* The lymphatic vessels take in all kinds of morbid poisons from external nature, when they come in contact with them. On this, rests the possibility of inoculation, infection by the bite of a rabid animal, by impure coition, &c.

*b.* They also take up from the interior of the body poisons produced therein; *e. g.* from carcinomatous ulcers: secondary syphilis seems to arise in this way.

*c.* They are inflamed only by some poisons, not at all by others, sometimes by others, and the vital properties of the poison contribute nothing to this effect. They are never inflamed by rabid poison; this may lie in them for whole months, without producing an effect, and yet this is one of the most frightful poisons.

*d.* The lymphatic vessels do not work any change on the poison that has been taken in, they do not assimilate it, but let it retain the same qualities it had when taken. This third or last assertion may require confirmation, and here it is: if any morbid poison be taken up from any other body by the lymphatic vessels, it remains inactive and without exciting any phenomena, so long as it lies in the lymphatic system. Perhaps it may excite inflammation of the lymphatic vessel filled with it, or of the gland next to which it lies. But as soon as it mixes with the blood, it produces the same disease as that under which the person now yielding the poison had suffered, whether the poison may have tarried a longer or shorter time in the lymphatic system of the person now infected. Were it in the slightest degree assimilated, another form of disease would break out, but not the same as the poison had produced in the other.

These, says our author, are four undeniable facts, which are still further confirmed by a fifth, viz. that a quantity of poison rubbed into a wound and into the skin will injure, whilst in the stomach it is entirely innoxious, a proof that the lymphatic system has not the power of assimilation, whilst the stomach has. But the question now comes, whether notwithstanding this, morbid poisons may not be generated in the lymphatic system? The accurate investigation of this question is attended with considerable difficulty. No one denies that diseases, the result of dyscrasia, may arise and actually do arise from bad chylification, as is proved by gout, scrophula, and other diseases; but whether this bad chylification has its origin in the intestinal canal and its secretions, or only in the absorbing vessels of the same, whether the visible disease of the latter is to be considered as originally in them or as a consequence of the morbid state of the intestines, that is the question not easily answered.

In every case it is certain, that the entire state of the lymphatic system is very considerably changed in scrofulous disease: the glands are broken up; the diameter of their vessels becomes increased, and the external lymphatic glands more especially swell, often pass into inflammation and suppuration, and, whether as a cause or consequence of the entire disease, still continues a question.

But if the qualitative changes in the action of the lymphatic system cannot be demonstrated with perfect certainty, and so the theory falls according to which this is considered as the principal source of the dyscrasiæ, yet it is certainly capable of quantitative changes in its action, nay it is at least extremely probable, that even an antagonism takes place between the lymphatic system of the intestinal canal and that of the rest of the body, whereby the former becomes more active, when the latter is less engaged, and vice versâ. Hungry persons, for instance, become more readily affected by infectious poisons, than persons satiated with food, and the dispersion of ecchymosis is promoted by purgatives and emetics. Absorption is much more active in youth than in adult age, especially that in the intestinal canal, whence, at least in part, arises the decline of nutrition in advanced years. The lymphatic vessels take no share in the diseases of the vascular system; they continue their functions uninterrupted in the midst of the most violent fevers, and according as the supply which should compensate the absorbed mass becomes diminished, they are the great causes of the emaciation which occurs in diseases of the blood-vessels. This want of participation in febrile diseases proves the low degree of vitality in the lymphatic system, if further proof were wanted.

We next come to the mucous membranes, the anatomical and physiological account of which we shall pass over for obvious reasons, and proceed to the pathological phenomena of this system, so far as it is an organ of secretion, or rather a system of secreting organs. Considered as such, the quantity of the secretion may first be at fault; it may be too copious, it may be too small, it may cease altogether. In the second place, the quality of the secretion may deviate from that which is natural, and in the third place, new organs may form for secretion in mucous membranes, or the relations of the parts of which mucous membranes consist may be so changed as to give the semblance of a new formation, the external appearance of the same assuming an unusual form.

The chief end of the secretion of the mucous membrane is obviously not the purification of the blood, nor the formation of a specific matter, nor the evacuation of any residue of parts which have reached the interior, but do not correspond to the purposes of life, but a sort of protection, which the mucous membrane finds necessary to its special destination as a mediating organ between the external world and the internal organism. The mucus accordingly compensates it for the want of the horny structure, which protects the skin, or it has it for the same purpose which this has for the skin. Only in some parts of this system, in the bronchial ramifications and the stomach especially, matters are somewhat otherwise, but then the secretion does not consist of mere mucus, and entirely different circumstances occur through the disturbance of these secretions, which appertain to the mucous system as such. Nay, we shall convince ourselves that the diseases of the stomach, bronchial system, and uterus, which depend on mere change of the mucous secretion, are of an entirely different nature and import from those which concern the proper secretions of these organs.

The quantity of the secretion of the mucous membrane is very unequal in the different periods of the life of man, and in certain individuals. In infants it is rather copious. In a subsequent period of childhood the mucous secretion is always more scanty, until a few years after the commencement of puberty it attains its minimum, whence there is at this age a greater disposition to hæmorrhages from the mucous membranes, simply because these are less protected than usual from the impressions of the atmosphere. At a later age they again gradually, but slowly, become more copious, till at a more advanced period they increase considerably, especially in the genito-urinary parts of the system, where they give rise to catarrhus vesicæ. So it occurs with the generality of persons, but there are many exceptions, in which the mucous secretion continues copious during the entire of life. Not rarely have the temperaments of individuals been determined by these differences; thus, a man with copious mucous secretion has been called phlegmatic, one with a very small quantity, melancholic, a mischievous error no doubt, but the natural consequence of the vacillating import attached to the term temperament.

It is morbidly diminished by two entirely opposite causes, viz. by increased contraction in the vessels of the mucous membranes and inflammation of the same, also by increased expansion. The first cause produces the natural diminution of the secretion in the adult age. The child's body is more relaxed than that of the adult; expansion predominates over contraction, and so it then happens that the secretion of the mucous membranes decreases with increasing maturity, in which the quality of contraction always gains the upper hand. At a more advanced period the imperfection of the process of oscillation and the diminished capability to assimilate that which comes from without act so that those secretions increase which compensate for the diminished serous secretions, which are considerably lessened by age, and in this way we may account for the increase of the mucous secretions which then occurs. The increased contraction of the vessels of the mucous membrane also produces a dryness of the same, for want of juices and of supply. But the mucous secretion in the membranes does not entirely cease, if they are inflamed: the stoppage of the exchange of matter in the minute vessels then renders all secretion impossible. If the inflammation be shared with the cellular membrane, by which the mucous membrane is connected with the subjacent parts, it dies and then presents a thick, white, wrinkled mass, which gradually detaches itself. The mucous membrane does not pass into suppuration, but it sometimes becomes covered with pus, viz. when the inflammation becomes superficial. Then one of two things may happen; either the membrane, at first entirely dry, presents places, which altogether resemble excoriations of the skin, which are covered with a little thin pus, or it resumes again the office of secretion, but after an entirely different manner from before; instead of mucus it secretes plastic lymph. The mucous membrane can, in fact, suppurate only when ulcers form in the subjacent parts; these perforate it. Hence we never see, for instance, syphilitic ulcers in the urethra or vagina. But they are of frequent occurrence in the mouth and throat with destruction of the mucous membrane, but never in this same membrane, but in the muscular or glandular structures lying beneath.

Increase of the mucous secretion occurs much more frequently than its diminution; it is the necessary consequence of every irritant which produces congestion of blood in the minute vessels of the mucous membranes. And so acts almost every irritant, at least at the commencement; for though the mucous membrane is accustomed to the contact of external matters, yet by their constant action and greater change their influence must be felt quite differently, and accordingly produce on the action of their vessels as important and frequent changes as in their nervous action. Thus life manifests itself variously at various periods in the mucous membranes, consequently the influx of blood into their vessels, from which their dilatation follows, is constantly subject to increase and diminution. The first have an increase of secretion as a natural consequence. Cold produces such an increase, most rapidly even in the parts of the mucous membrane most exposed to it, in the nose, eyes, throat, and air-passages. Every one who goes into the cold will soon perceive this, by the moisture of his nose; the discharge which the cold produces, and the moist state of the eye, prove it. Cold also produces copious mucous secretion in the throat, witness the coldness of ice in Summer; it does less injury in the stomach, and in the intestines also, in which cold injections are often beneficial. But throughout the entire genito-urinary system of mucous membranes cold will not only prove very serviceable, but will even check the profuse mucous secretion, when it arises from other causes. Cold acts on the bladder, the genitals, and even on the stomach itself, as a very violent, unaccustomed stimulus on the sensible nervous surfaces, and its action on the vascular net-work of the mucous membranes does not promote the secretion ; it rather exalts the entire vitality of the organ. On the contrary, in the air-passages and the eyes, it is a usual stimulus, which affects the nervous action less, and excites the secretion much more.

We shall defer further notice of this work till our next number. As far as we have proceeded in our analysis of it, we have no hesitation in saying that it approximates more nearly to our idea of what a work on General Pathology should be than any we have yet seen. The author is decidedly a pathologist of the anatomical school, and has accordingly produced a book replete with sound practical information, available in the diagnosis and treatment of disease as it occurs in every-day practice.

